# Beyond conventional wisdom: unexplored risk factors for penile fracture

**DOI:** 10.1093/sexmed/qfae068

**Published:** 2024-10-01

**Authors:** Mehmet Gürkan Arikan, Burak Akgul, Semih Turk, Ömer Onur Tantekin, Basri Çakiroğlu, Ersan Arda

**Affiliations:** Department of Urology, Hatay Dörtyol State Hospital, Hatay 31600, Turkey; Department of Urology, Trakya University School of Medicine Hospital, Edirne 22000, Turkey; Department of Urology, Edirne Sultan 1. Murat State Hospital, Edirne 22000, Turkey; Sisli Hamidiye Etfal Training and Research Hospital Department of Urology, Sisli 34360, Turkey; Department of Urology, Istanbul Galata University, Hisar Intercontinental Hospital, Istanbul 31600, Turkey; Department of Urology, Trakya University School of Medicine Hospital, Edirne 22000, Turkey

**Keywords:** penile fracture, anthropometric parameters, sexual positions, urethral involvement, body mass index, traumatic episodes, partner’s weight, medial lesions, multicenter study, sexual health

## Abstract

**Background:**

Sexual intercourse is a predominant cause of penile fractures (PF).

**Aim:**

To evaluate the relationship between body metrics and the severity of PF, identifying how anthropometric factors of patients and their partners influence the incidence and severity of PF.

**Methods:**

This study analyzed 47 patients with PF over 9 years across multiple centers. Demographic and anthropometric data were collected for both patients and their partners. Injuries were categorized by the sexual position or activity causing them, including partner-related positions (man-on-top, woman-on-top, and doggy-style) and isolated actions (rolling-over, blunt trauma, masturbation, or manipulation). Statistical analysis was performed using the χ2-test.

**Outcomes:**

The study aimed to uncover correlations between body metrics and the risk profile for PF, focusing on body mass index (BMI), weight, height, and age.

**Results:**

The majority of PFs occurred during sexual activities (74.5%), particularly in “doggy” and “man-on-top” positions. Urethral involvement was observed in 10.6% of cases. Significant correlations were found between patient and partner ages (*r* = 0.915), patient BMI and weight (*r* = 0.874), patient height and weight (*r* = 0.502), and partner weight and BMI (*r* = 0.805). Higher BMI in patients was linked to delayed hospital visits (*r* = 0.377), and frequent sexual activity was strongly correlated with urethral complications (*r* = 0.727).

**Clinical Implications:**

These findings emphasize the importance of considering body metrics in assessing PF risk and could guide future preventive strategies and clinical interventions.

**Strengths & Limitations:**

The study’s strength lies in its comprehensive analysis of anthropometric factors and their correlation with PF severity. Limitations include a relatively small sample size and the potential for reporting bias in sexual activity data.

**Conclusion:**

The study confirms sexual intercourse as the main cause of PF and highlights significant links between anthropometric factors and fracture severity. Higher BMI and weight in partners are associated with more centrally located fractures and urethral injuries, particularly in taller patients or those with larger partners.

## Introduction

Penile fractures (PF) are a critical urological emergency characterized by rupture of the tunica albuginea of the corpora cavernosa, often requiring immediate medical intervention [1]. Such fractures are uncommon, with incidences reported as low as one in every 175 000 cases in the United States and between 1.14 and 10.48 per 100 000 men in East Asia [2]. While the primary treatment often consists of swift surgical intervention, the absence of timely and proper management may lead to several adverse outcomes, such as impaired erectile function, penile deformity, and scar tissue formation [3].

Given the sensitive and private nature of the circumstances leading to PF, there is often reluctance in thorough reporting and a consequent gap in comprehensive research [4]. Sexual intercourse, the most common cause of PF, is influenced by a myriad of social and personal dynamics, making it imperative to consider these factors when addressing injuries [5]. Several studies have highlighted the impact of anthropometric parameters such as penile length, girth, and curvature on the risk of PF. However, the specific nuances of body metrics remain unexplored [6]. The critical nature of PF necessitates a thorough grasp of all contributing elements to improve patient care. There remains a paucity of literature examining the potential influence of body dimensions and anthropometric parameters on the incidence and severity of these injuries [7, 8].

Moreover, the current literature falls short of exploring how patient and partner anthropometry interplay with the risk and severity of the condition [9, 10]. Understanding the risks of sexual positions and related factors is essential for improving sexual health and preventing injuries like PF. This study explores the link between body metrics and the severity of PF, focusing on the Turkish population to tailor interventions to specific demographics. We hypothesize that body dimensions, such as body mass index (BMI) and weight, influence both the cause and severity of these injuries, impacting their management.

This study aimed to bridge the existing knowledge gap by exploring the impact of anthropometric parameters on the occurrence and severity of PF. By examining these relationships, we aspire to enhance the understanding of PF within the scope of sexual health, offering new perspectives on risk assessment and contributing to the refinement of clinical practices for better patient care and outcomes.

## Methods

We conducted a 9-year multicenter retrospective cohort study from January 2013 to December 2021 on 47 male patients with clinically diagnosed PF, approved by institutional review boards and adhering to the Declaration of Helsinki. Inclusion criteria were men aged ≥18 years diagnosed via ultrasonography or MRI; exclusions were non-consent, incomplete data, and non-sexual injury causes. Originally, non-sexual causes as exclusion criteria referred to traffic accidents and falls, and this has been revised in the material and method section. The non-sexual activities (25.5%) included in the study refer to fractures occurring during rolling over and manual manipulation. Data on demographics, sexual orientation, medical history, and anthropometric measurements (height, weight, BMI) were collected. Injury mechanisms were categorized as partner-related or self-manipulation, with fractures classified by location and urethral involvement. Statistical analyses included descriptive statistics, χ2-test, Fisher’s exact test, and Pearson’s correlation, using SPSS software (version 25.0). Ethical approval was granted by XX Committee, with strict adherence to confidentiality and informed consent protocols.

## Results

### Demographic and anthropometric characteristics

The study cohort comprised of 47 male patients with sustained PF. The mean age of the participants was 39.6 years (SD = 12.964) years, ranging from 24 to 71 years. The mean height was recorded at 175.34 centimeters (cm) (SD = 5.828 cm), with the shortest and tallest participants measuring 160 and 192 cm, respectively.

The mean age of the partners involved was 36.55 years (SD = 11.086), ranging from 21 to 61 years. The average height of the partners was 161.06 cm (SD = 5.435 cm), with a range extending from 150 to 172 cm. The mean weight was found to be 63.57 kilograms (kg) (SD = 8.179), with a spectrum ranging from 48 to 80 kg. The partners’ BMI averaged 24.4321 (SD = 2.96556), where the lowest recorded BMI was 18.29, and the highest was 31.2, indicating a range from normal to overweight according to the World Health Organization’s BMI classification.

#### Comorbid conditions

Most of the patients did not have any additional comorbid conditions, with 87.2% (n = 41) reporting no coexisting diseases. Diabetes mellitus (DM) was present in 4.3% (n = 2) of the participants, while 6.4% (n = 3) reported hypertension (HT). Only one patient (2.1%) had a combination of diabetes and hypertension (DM + HT).

#### Sexual intercourse duration and frequency

Regarding the time elapsed from the injury to presentation at the medical facility, there was a median of 6 h (SD = 26.855), with a minimum time of 1 h and the maximum recorded at 144 h.

The duration of sexual intercourse during which the injury occurred had a mean value of 18.43 minutes (SD = 9.571), ranging from 2 to 60 minutes. The median of sexual intercourses per week reported by the patients was 2 (SD = 1.525), with a minimum of 1 and a maximum of 7 times per week.

#### Features and mechanisms of trauma

Out 47 cases of PF, a large majority (74.5%, n = 35) were attributed to activities during sexual intercourse. The remaining 25.5% (n = 12) of the cases were unrelated to sexual activity. Analyzing the sexual positions at the time of injury, the “doggy” position was the most common, associated with 29.8% (n = 14) of the fractures. This was followed by the “man-on-top” position, which was implicated in 21.3% (n = 10) of the cases. The “woman-on-top” and “rolling” positions were each associated with 17% (n = 8) of the fractures. Manual manipulation was the cause of 10.6% (n = 5) of the incidents, and blunt trauma was the least common mechanism, accounting for only 4.3% (n = 2) of cases.

Urethral involvement was observed in 10.6% (n = 5) of patients with PF, while the majority (89.4%, n = 42) did not have any urethral injury associated with the fracture.

### Comparative analyses of study parameters

The lesion location relative to the presence of a partner during the injury revealed a statistically significant association with proximal PF (*P* = 0.048), where 90% of such injuries occurred during intercourse with a partner. This correlation was not observed in the midshaft or distal fractures.

Table 1 presents the examination of lesion location relative to the presence of a partner during injury.

**Table 1:** Examination of lesion location relative to the presence of a partner during injury.

**Lesion location**	**With partner (Count, %)**	**Without partner (Count, %)**	**Total (Count)**	***P*-value**
Proximal	18 (90.00%)	2 (10.00%)	20	**0.048**
Midshaft	16 (94.12%)	1 (5.88%)	17	-
Distal	8 (80.00%)	2 (20.00%)	10	-
Right	25 (89.29%)	3 (10.71%)	28	0.984
Left	17 (89.47%)	2 (10.53%)	19	-

-: *P*-values are not applicable or not calculated due to the small sample size in the subgroups.

*P*- Fisher’s Exact Test.

Values with *p* < 0.05 were considered statistically significant and are indicated in bold.

It also demonstrates the relationship between penile fracture location and partner presence at the time of injury according to lateralization. Lateralization of the injury (right or left) showed no significant correlation with partner presence (*P* = 0.984).

Table 2 presents the patients’ anthropometric and sexual health parameters in relation to lesion location in detail.

**Table 2:** Patients’ anthropometric and sexual health parameters in relation to lesion location in detail.

**Parameter**	**Lesion location**	**N**	**Mean**	**Std. Deviation**	**Minimum**	**Maximum**	***P*-value**
**Patient’s**							
**Height (cm)**	Proximal	20	174.15	4.17	165	182	0.215
	Midshaft	17	175.12	6.31	160	189	-
	Distal	10	178.10	7.37	165	192	-
**Weight (kg)**	Proximal	20	74.80	9.14	55	91	0.158
	Midshaft	17	81.12	10.27	65	105	-
	Distal	10	75.90	11.55	62	92	-
	Total	47	77.32	10.29	55	105	-
**BMI**	Proximal	20	24.68	2.84	17.96	29.4	0.052
	Midshaft	17	26.54	3.46	20.52	33.2	-
	Distal	10	23.87	2.87	19.57	27.46	-
Partner’s							
**Height (cm)**	Proximal	20	160.30	6.77	150	172	0.625
	Midshaft	17	162.06	3.78	155	170	-
	Distal	10	160.90	5.04	155	170	-
**Weight (kg)**	Proximal	20	63.35	8.08	48	80	**0.049**
	Midshaft	17	66.59	8.18	50	80	-
	Distal	10	58.90	6.59	49	70	-
**BMI**	Proximal	20	24.56	2.70	18.29	29.6	0.104
	Midshaft	17	17	25.25	3.18	18.3	-
	Distal	10	22.76	2.65	19.33	27.24	-
**Duration of intercourse (min)**	Proximal	20	17.8	7.72	2	30	0.903
	Midshaft	17	18.53	13.08	5	60	-
	Distal	10	19.5	5.98	10	30	-
**Weekly frequency of intercourse**	Proximal	20	1.95	0.999	1	4	**0.047**
	Midshaft	17	2	1.696	1	7	-
	Distal	10	3.3	1.767	1	7	-

-: *P*-values are not applicable or not calculated due to the small sample size in the subgroups.

*P*- Kruskal–Wallis test.

There was no significant difference in the height of the patients across different lesion locations. The patient’s weight and BMI did not differ significantly by lesion location; although proximal fractures tended to be associated with lower weights and BMIs than midshaft fractures, this did not reach statistical significance. The partner anthropometric measures were not significantly different across various fracture locations. The duration and weekly frequency of intercourse showed no significant variation with fracture location. A significant relationship was found between the weekly frequency of intercourse and proximal PF (*P* = 0.047).

Table 3 presents a detailed breakdown of the occurrence of urethral involvement in PF, differentiated by the location of the lesion and activity at the time of injury.

**Table 3:** The detailed breakdown of the occurrence of urethral involvement in penile fractures, differentiated by the location of the lesion and activity at the time of injury.

Lesion location	Urethral involvement (Count, %)			
	**No**	**Yes**	**Total (Count)**	***P*-value**
Proximal	16 (80.00%)	4 (20.00%)	20	0.479
Midshaft	16 (94.12%)	1 (5.88%)	17	-
Distal	10 (100.00%)	0 (0.00%)	10	-
Right	25 (89.29%)	3 (10.71%)	28	0.984
Left	17 (89.47%)	2 (10.53%)	19	
**Activity Nature**				
With partner	38 (90.48%)	4 (9.52%)	42	**0.048**
Without partner	4 (80.00%)	1 (20.00%)	5	-

-: *P*-values are not applicable or not calculated due to the small sample size in the subgroups.

*P*- Chi-square (χ2) test.

The analysis showed no significant link between lesion location and activity during injury and urethral involvement in PF. However, fractures near the base had more urethral involvement, especially during sexual intercourse with a partner.

### Correlation analyses of study parameters

Table 4 shows the demographic, anthropometric, and clinical variables in penile fracture patients. There was a particularly strong positive correlation between the patient’s age and partner’s age (*r* = 0.915, *P* < 0.05), and there was a significant positive correlation between the patient’s weight and BMI (*r* = 0.874, *P* < 0.05). Patient height showed a moderate positive correlation with weight (*r* = 0.502, *P* < 0.05), and partner weight was significantly correlated with BMI (*r* = 0.805, *P* < 0.05). In terms of clinical variables, the time to hospital presentation was positively correlated with the patients’ BMI (*r* = 0.377, *P* < 0.01), suggesting that individuals with a higher BMI may experience delays in presenting to the hospital.

**Table 4:** The Spearman’s correlation analysis of demographic, anthropometric, and clinical variables in penile fracture patients.

**Variable**	**Patient age**	**Height (cm)**	**Weight (kg)**	**BMI**	**Partner’s Age**	**Partner’s height (cm)**	**Partner’s weight (kg)**	**Partner’s BMI**	**Duration of relationship**	**Weekly frequency of intercourse**	**Time to presentation (hours)**	**Urethral involvement**
**Patient age**	r = 1	r = -0.258	r = 0.052	r = 0.202	**r = 0.915** ^**b**^	r = -0.046	r = 0.251	r = 0.282	r = −0.278	r = −0.128	r = 0.274	r = −0.378
	*P* = .	*P* = 0.08	*P* = 0.728	*P* = 0.173	***P* = 0.041**	*P* = 0.761	*P* = 0.089	*P* = 0.055	*P* = 0.059	*P* = 0.391	*P* = 0.062	*P* = 0.159
**Height (cm)**	r = -0.258	r = 1	**r = 0.502** ^**a**^	r = -0.14	r = -0.178	r = 0.223	r = -0.003	r = -0.089	r = 0.028	r = 0.203	r = -0.085	r = 0.022
	*P* = 0.08	*P* = .	***P* = 0.039**	*P* = 0.347	*P* = 0.231	*P* = 0.132	*P* = 0.982	p = 0.55	*P* = 0.852	*P* = 0.172	*P* = 0.572	*P* = 0.652
**Weight (kg)**	r = 0.052	**r = 0.502** ^**a**^	r = 1	**r = 0.874** ^**b**^	r = 0.098	r = 0.232	r = 0.226	r = 0.179	r = 0.029	r = -0.015	r = 0.096	r = -0.128
	*P* = 0.728	***P* = 0.039**	*P* = .	***P* = 0.032**	*P* = 0.511	*P* = 0.116	*P* = 0.126	*P* = 0.23	*P* = 0.847	*P* = 0.918	*P* = 0.523	*P* = 0.391
**BMI**	r = 0.202	r = -0.14	**r = 0.874** ^**b**^	r = 1	r = 0.211	r = 0.134	r = 0.221	r = 0.203	r = -0.004	r = −0.124	r = 0.128	r = 0.203
	*P* = 0.173	*P* = 0.347	***P* = 0.032**	*P* = .	*P* = 0.155	*P* = 0.370	*P* = 0.136	*P* = 0.17	*P* = 0.976	*P* = 0.407	*P* = 0.391	*P* = 0.172
**Partner’s age**	**r = 0.915** ^**b**^	r = -0.178	r = 0.098	r = 0.211	r = 1	r = −0.007	r = 0.629^a^	**r = 0.428** ^**a**^	r = −0.144	r = −0.187	r = 0.289^a^	r = 0.045
	***P* = 0.041**	*P* = 0.231	*P* = 0.511	*P* = 0.155	*P* = .	*P* = 0.961	*P* = 0.024	***P* = 0.025**	*P* = 0.335	*P* = 0.208	*P* = 0.049	*P* = 0.049
**Partner’s height**	r = -0.046	r = 0.223	r = 0.232	r = 0.134	r = −0.007	r = 1	**r = 0.605** *	r = −0.145	r = 0.156	r = -0.031	r = -0.088	r = 0.118
**(cm)**	*P* = 0.761	*P* = 0.132	*P* = 0.116	*P* = 0.37	*P* = 0.961	*P* = .	***P* = 0.005**	*P* = 0.33	*P* = 0.295	*P* = 0.834	*P* = 0.555	*P* = 0.407
**Partner’s Weight**	r = 0.251	r = -0.003	r = 0.226	r = 0.221	**r = 0.629** ^**a**^	**r = 0.605** ^**b**^	r = 1	**r = 0.805** ^**b**^	r = −0.112	r = −0.298^a^	r = 0.294^a^	r = 0.118
**(kg)**	*P* = 0.089	*P* = 0.982	*P* = 0.126	*P* = 0.136	***P* = 0.024**	***P* = 0.005**	*P* = .	***P* = 0.037**	*P* = 0.453	*P* = 0.042	*P* = 0.045	*P* = 0.118
**Partner’s BMI**	r = 0.482	r = -0.089	r = 0.179	r = 0.203	**r = 0.428** ^**a**^	r = -0.145	**r = 0.805** ^**b**^	r = 1	r = -0.178	r = -0.227	r = 0.377^b^	r = 0.407
	*P* = 0.055	*P* = 0.55	*P* = 0.230	*P* = 0.17	***P* = 0.025**	*P* = 0.33	***P* = 0.037**	*P* = .	*P* = 0.23	*P* = 0.124	*P* = 0.009	*P* = 0.429^a^
**Duration of**	r = −0.278	r = 0.028	r = 0.029	r = −0.004	r = −0.144	r = 0.156	r = -0.112	r = −0.178	r = 1	r = 0.086	r = −0.172	**r = 0.728** ^**b**^
**relationship**	*P* = 0.059	*P* = 0.852	*P* = 0.847	*P* = 0.976	*P* = 0.335	*P* = 0.295	*P* = 0.453	*P* = 0.23	*P* = .	*P* = 0.567	*P* = 0.248	***P* = 0.035**
**Weekly Frequency**	r = -0.128	r = 0.203	r = -0.015	r = -0.124	r = -0.187	r = −0.031	**r =** −0.298*	r = -0.227	r = 0.086	r = 1	r = −0.181	**r = 0.727** ^b^
**of Intercourse**	*P* = 0.391	*P* = 0.172	*P* = 0.918	*P* = 0.407	*P* = 0.208	*P* = 0.834	***P* = 0.042**	*P* = 0.124	*P* = 0.567	*P* = .	*P* = 0.222	***P* = 0.039**
**Time before**	r = 0.289^a^	r = -0.085	**r = 0.294** ^**a**^	**r = 0.377** ^**b**^	r = 0.274	r = -0.088	r = 0.096	r = 0.128	r = -0.172	r = -0.181	r = 1	r = 0.242
**admission (hours)**	*P* = 0.049	*P* = 0.572	***P* = 0.045**	***P* = 0.009**	*P* = 0.062	*P* = 0.555	*P* = 0.523	*P* = 0.391	*P* = 0.248	*P* = 0.222	*P* = .	*P* = 0.572
**Urethral**	r = -0.378	r = 0.022	r = -0.128	r = 0.203	r = 0.918	r = 0.118	**r = 0.629** ^**a**^	**r = 0.631** ^**a**^	**r = 0.728** ^**b**^	**r = 0.727** ^**b**^	r = 0.242	r = 1
**Involvement**	*P* = 0.159	*P* = 0.652	*P* = 0,391	*P* = 0.172	*P* = 0,407	*P* = 0.407	***P* = 0.045**	***P* = .049**	***P* = 0.035**	***P* = 0.039**	*P* = 0.572	*P* = .

a indicates *P* < 0.05, suggesting a statistically significant correlation.

b indicates *P* < 0.01, suggesting a highly statistically significant correlation.

N = Number of observations (47 for all variables, and 5 for the urethral involvement)

Abbreviation: BMI, body mass index

The weekly frequency of intercourse showed a strong positive correlation with urethral involvement (*r* = 0.727; *P* < 0.05). The duration of the sexual intercourse was inversely correlated with the patient’s age (*r* = −0.278, *P* = 0.059), although this did not reach statistical significance. The strongest negative correlation was observed between patient age and urethral involvement (*r* = −0.378, *P* = 0.159), but this correlation was not statistically significant.

## Discussion

This comprehensive study aimed to examine a wide array of anthropometric parameters and their association with PF in the Turkish population. By elucidating the complex interplay between anthropometric parameters and PF, our findings may pave the way for more targeted education and awareness campaigns as well as the development of personalized preventive strategies. This research has the potential to significantly impact the fields of urology and public health, offering valuable insights that could ultimately enhance the well-being of individuals within the Turkish population and beyond.

In examining the demographic and anthropometric characteristics of the study cohort, the mean age closely matched that reported in broader research, indicating the prevalence of PF among middle-aged patients [11]. The encompassed age ranges from young to older adults highlight the study’s widespread relevance, affirming its utility across varying age groups. Additionally, the cohort’s height and weight distributions align with global standards, reinforcing the applicability of this study to a diverse array of populations [12, 13]. The infrequency of comorbid conditions such as diabetes and hypertension in the cohort echoes findings from the existing literature, suggesting a lack of correlation between PF and these conditions [14, 15]. This trend towards the predominance of patients without significant comorbidities emphasizes that acute mechanical trauma, rather than pre-existing chronic diseases, serves as the primary catalyst for PF. This revelation is crucial, as it delineates the risk of penile fracture as predominantly tied to immediate traumatic incidents, rather than the broader health profile of the affected individuals [14, 16].

The median delay of 6 h before seeking medical help post-injury, as observed in this study, coupled with a wide variability in response times, underscores a significant gap in public awareness regarding the severity of PF. This delay in seeking medical attention may be due to various factors, such as embarrassment or a lack of awareness about the seriousness of the injury [17]. This calls for intensified public health campaigns to educate patients on the urgency of prompt treatment to prevent further complications [18].

The findings on the average duration and median frequency of sexual activities preceding the injury fall within the expected parameters established by previous studies [10], which suggests that neither the length nor the frequency of sexual intercourse significantly modifies the risk of PF. The role of sexual intercourse, particularly in “doggy” and “man-on-top” positions, as a leading cause of PF is corroborated by a recent meta-analysis [10], highlighting the increased risk associated with these sexual practices. Furthermore, the complication rates of urethral involvement presented herein are consistent with those documented in the literature [2]. This study reinforces the necessity of recognizing high-risk sexual behaviors and underscores the vital role of patient education regarding the inherent risks linked to certain sexual positions. The concordance between the frequency results of this study and extant research strengthens the argument for immediate medical assessment and intervention following PF to reduce the risk of enduring consequences.

A strong positive correlation between patient age and partner age (*r* = 0.915, *P* = 0.041) suggested that PF were consistent across similar age groups for couples. This could be indicative of sexual habits or preferences within a similar age demographic or could suggest that couples of similar ages may engage in sexual activity with a similar frequency or intensity.

In the context of the study’s findings on the strong positive correlation between patient and partner’s age in PF, the literature suggests that sexual practices and preferences, which may be influenced by age, contribute significantly to the incidence of PF [19]. While specific studies directly correlating partners’ age groups with penile fracture risk are limited, research indicates that sexual trauma is the main etiological factor in PF, which could conceivably be influenced by age-related sexual behaviors. Barros et al. examined the outcomes of penile fracture patients and provided comparative data on the demographics of such injuries, although it did not directly address the age correlation between partners [20].

Partner’s weight showed a strong positive correlation with their BMI (*r* = 0.805, *P* = 0.037) and a moderate positive correlation with the patient’s weight (*r* = 0.629, *P* = 0.024). The clinical implications of these findings should be discussed, such as the role of the partner’s physique in the mechanics of sexual intercourse that may contribute to the injury. Regarding the partner’s anthropometric measures, the literature is more focused on the physical mechanisms of injury and the positions leading to PF rather than the partners’ body metrics. However, it is understood that the it “woman-on-top” position is associated with a higher risk of PF, suggesting that partners’ movements and control during intercourse could play a role in the mechanism of injury [20, 21]. The relationship between a partner’s weight, BMI, and penile fracture has not been extensively studied; however, the mechanics of sexual intercourse and the risk of injury could logically be influenced by these factors.

A negative correlation between the duration of the sexual activity and weekly frequency of intercourse (*r* = −0.298, *P* = 0.042) may suggest that sexual frequency decreases over time in a relationship, which could have implications for sexual health and counseling. Regarding the relationship between duration and sexual frequency, there are indications that PF might occur when sex is rushed or in unusual locations, which could correlate with the novelty of sexual relationships or extramarital affairs. Majzoub et al. and Bali et al. found that half of patients with PF experienced injury during an extramarital affair, suggesting that relationship context and sexual frequency or intensity could be influential [6, 22].

The time before admission was moderately correlated with urethral involvement (*r* = 0.377, *P* = 0.009), indicating that delays in seeking medical care after penile trauma might lead to more severe injuries, including urethral damage. This underscores the importance of prompt medical attention to prevent severe complications, as supported by literature emphasizing immediate treatment to avoid long-term consequences.

The study identified strong positive correlations between urethral involvement and both the weekly frequency of intercourse (*r* = 0.727, *P* = 0.039) and the duration of sexual activity (*r* = 0.728, *P* = 0.035). These results indicate that higher frequency and longer durations of sexual activities may increase the risk of urethral injury during PF. This suggests that certain sexual practices, possibly evolving over time in a relationship, could be linked to the severity of injuries, consistent with other findings that specific sexual positions and scenarios vary in their risk of penile and urethral injuries [22, 23].

To provide context for our findings and highlight similarities or differences, we extensively reviewed the existing literature. Tolani et al. [18] discussed the demographic burden and clinical patterns of penile trauma across different age groups, confirming that penile trauma predominantly affects active adults, consistent with our findings. However, their study did not explore the impact of anthropometric factors. Syarif et al. [10] identified the “doggy style” and “man-on-top” positions as the most dangerous for causing PF, which aligns with our findings that these positions are associated with more severe fractures. Rahman et al. [24] noted that penile manipulation is a common cause of fractures in the Middle East, similar to our findings where manipulation also played a significant role. Barros et al. [20] examined how sexual positions affect the severity of fractures, finding that the “doggy style” position leads to more severe injuries, which is consistent with our results. Oranusi and Nwofor [25] emphasized the challenges in managing severe penile injuries and the importance of early surgical intervention, aligning with our findings that higher BMI leads to delayed treatment. Nawaz et al. [26] highlighted the importance of early surgical repair for PF, which our study supports by showing that delays in seeking medical care due to higher BMI can lead to more severe injuries. Majzoub et al. [6] reported that vigorous sexual intercourse is the most common cause of PF in the Middle East and Central Asia, which is also observed in our study within the Turkish population. Rodriguez et al. [27] noted healthcare access disparities affecting penile fracture outcomes in the US, and our study similarly underscores the importance of public health awareness to reduce treatment delays. Baylan et al. [28] found that female sexual dysfunction factors such as vaginal dryness and dyspareunia significantly contribute to PF, while our study highlights the correlation between partner weight and BMI and fracture risk. This comparative analysis underscores the novel contribution of our study in understanding the multifaceted nature of penile fracture risk and severity, emphasizing the need to integrate anthropometric assessments into clinical evaluations and public health strategies to improve patient outcomes and preventive measures.

## Study limitations and future research aide

The study faced several limitations, including a small sample size that limits generalizability to a broader population, its retrospective nature introducing potential recall bias, and a focus on the Turkish population which may not reflect wider behavioral patterns due to cultural differences. Additionally, data based on self-reported accounts may be unreliable given the sensitive topic. The study was designed retrospectively, with patients being contacted via telephone. The central city of the study is a socio-culturally advanced area. We did not encounter issues of embarrassment or biased responses when we explained the scientific benefit and concept of the study. Furthermore, patients who provided incomplete or inconsistent information were excluded from the study. Therefore, we believe there is no significant bias in the data. Future research should consider larger, more diverse cohorts and explore the efficacy of educational interventions and interdisciplinary approaches to better understand and manage PF, ultimately enhancing patient care and preventive strategies.

## Conclusion

This study delineated significant correlations between anthropometric measurements and the incidence of PF within the Turkish population, providing novel insights that could transform current urological and public health strategies. Identifying a strong positive correlation between patient age and partner age underscores the need for demographic-specific sexual health education and awareness programs. Our findings highlight the clinical relevance of partners’ anthropometric dimensions, establishing a noteworthy association between partners’ weight and BMI and the occurrence of PF and urethral injuries. The observed delay in seeking medical assistance and its positive correlation with urethral involvement highlights a critical gap in public awareness, emphasizing the need for immediate medical consultation following penile trauma. Integrating anthropometric evaluations into routine clinical practice can lead to personalized and effective prevention strategies, ultimately improving patient outcomes. The strong correlations identified between sexual positions and the severity of PF suggests the need for specific guidance on safer sexual practices. Overall, our study enhances the understanding of the multifaceted nature of penile fracture risk factors and solidifies the imperative for behavior-aware sexual education and trauma prevention, which could significantly reduce the incidence and severity of PF and improve sexual health and quality of life. Future research should focus on expanding these findings across diverse populations and exploring the long-term benefits of integrating anthropometric data into clinical and educational practices.
